# Left ventricular pacing in patients with preexisting tricuspid valve disease

**DOI:** 10.1002/joa3.12257

**Published:** 2019-11-11

**Authors:** Tony Y. W. Li, Swee Chong Seow, Devinder Singh, Wee Tiong Yeo, Pipin Kojodjojo, Toon Wei Lim

**Affiliations:** ^1^ Department of Medicine National University Hospital Singapore; ^2^ National University Heart Centre, Singapore (NUHCS) National University Hospital Singapore

**Keywords:** left ventricular pacing, pacemaker, tricuspid regurgitation, tricuspid valve

## Abstract

**Background:**

Conventional right ventricular (RV) pacing is increasingly recognised to cause tricuspid valve (TV) injury or dysfunction, in part due to the need to pass the lead through the valve. This may be especially problematic in patients with preexisting TV disease or prior TV surgery. An alternative in this situation is to implant a left ventricular (LV) lead instead of ventricular pacing.

**Methods:**

We performed a single‐center retrospective analysis of 26 patients with tricuspid valve surgery/disease who received a LV pacing lead in the coronary veins to avoid crossing the tricuspid valve, with or without a right atrial lead. A matched control population was obtained from patients receiving conventional right ventricular pacing and outcomes were compared. Main outcomes of interest were lead stability, electrical lead parameters and change in echocardiographic parameters such as left ventricular ejection fraction (LVEF) during long‐term follow‐up.

**Results:**

Successful left ventricular pacing was established in 25 out of the 26 cases with one case converted to a RV lead due to lead dislodgement. During the 2.96 ± 1.0 year follow‐up, 24 of 25 (96.0%) leads were functional with stable pacing and sensing parameters, and 1 of 25 (4.0%) was extracted for due to device infection following an episode of thrombophlebitis.

**Conclusion:**

We conclude that in patients with existing tricuspid valve disease or surgery, ventricular pacing via the coronary veins is a feasible, safe, and reliable alternative to right ventricular pacing.

## Abstract 2

We conducted a single center retrospective analysis of patients with tricuspid valve surgery or disease who received a LV pacing lead. Results show that in patients with existing tricuspid valve disease or surgery, ventricular pacing via the coronary veins is a feasible, safe and reliable alternative to right ventricular pacing.

## 1 INTRODUCTION

Implantable pacemakers are widely used in the treatment of patients with bradyarrhythmias and the conventional approach has been to implant leads into the right ventricle (RV), most commonly at the RV apex. The RV apex is often the site of choice for ventricular pacing given the relative ease and safety of implantation, low risk of displacement and good reliability.^1^ However, conventional RV pacing is increasingly recognised to cause tricuspid valve (TV) injury or dysfunction, in part due to the need to pass the lead through the valve.^2^, ^3^, ^4^, ^5^, ^6^ This is thought to occur through several different mechanisms including direct mechanical trauma and damage to the valves or due to interference with the valvular mechanism and may be especially problematic in patients with preexisting TV disease or prior TV surgery.^7^ Furthermore, long‐term right ventricular pacing has been shown to be deleterious to left ventricular function in some patients as it induces left ventricular dyssynchrony and can result in heart failure.^8^

There has been various experiences with patients in whom transvalvular leads are contraindicated, most prominently in patients with a mechanical tricuspid valve. In recent years there has been greater experience with LV pacing due to the advent of biventricular pacemakers or defibrillators and the use of contemporary left ventricular leads which are placed in the coronary veins in such devices.^9^, ^10^ In particular, the increasing use of cardiac resynchronization therapy device implants which involves LV lead implantation in the coronary veins has shown that LV pacing can be performed with low rates of lead dislodgement.^11^ This suggests the potential utility of this approach in patients with existing tricuspid valve pathologies.

In this report, we reviewed our experience with patients with tricuspid valve disease or prior surgery who required pacemaker implantation in whom a standard LV pacing lead was used in place of a conventional RV lead to demonstrate the safety and feasibility of this approach. Outcomes were compared to a matched group of patients with a conventional RV lead.

## 2 METHODS

A retrospective review of 26 consecutive patients who underwent pacemaker implantation with an LV lead from December 2012 to December 2015 was conducted. These patients were undergoing pacemaker implantation for standard indications but also had significant tricuspid valve disease (defined as moderate or severe regurgitation), or previous tricuspid valve surgery. They were all implanted with a standard single or dual chamber pacemaker with a bipolar LV pacing lead in place of a conventional RV lead. Procedures were all performed under local anaesthesia and with light sedation. The choice of the left ventricular lead and the vein targeted for implantation was left to the discretion of the operator. The patients were then followed as per usual clinical practice with pacemaker checks on 1 day, 1 week, 1 month, and 6 months after implantation. Subsequent follow‐ups were at yearly intervals or at the physician's discretion. Post implant transthoracic echocardiography was also performed to monitor for any change in the left ventricular function and for valvular pathology.

A set of 1:1 matched controls were then obtained from the institution pacemaker implant database during the same time period (2012‐2015) from patients receiving a conventional RV pacing lead. The controls were obtained by matching within the database for several key parameters notably: (a) age (less than 3 years age difference at time of device implantation), (b) gender, (c) less than 10% difference in left ventricular ejection fraction (LVEF) as well as (d) the type of pacemaker implanted (single or dual chamber). The first case that matched the above definitions with the procedural date closest to the original case was selected for comparison.

Key parameters assessed included lead interrogation parameters as well as echocardiographic variables at baseline and at multiple points during follow‐up. For both groups of patients, transthoracic echocardiography was performed prior to implantation. Follow‐up evaluation time‐points were at 6 months as well as the last available follow‐up through December 2017. We noted any procedural complication, any lead‐related problems during follow‐up, need for revision or abandonment of the implanted electrode, and date of death if the patient died. The severity of valvular regurgitation was routinely reported in the clinical echocardiographic studies based on American Society of Echocardiography recommendations.^12^

The two groups were compared for multiple parameters that were monitored over the duration of follow‐up where the primary outcome was lead stability over time. Categorical variables were compared using the chi‐square or Fisher exact test. Within‐group comparison was undertaken using Mann‐Whitney tests for echocardiographic data or repeated measures ANOVA testing for electrophysiologic data. A *P* < .05 was considered the threshold for statistical significance for all analyses. All statistical analyses were performed using SPSS Version 22 (IBM Corp.).

## 3 RESULTS

A conventional bipolar LV lead was implanted as part of a dual chamber (n = 14) or single chamber (n = 12) pacing system. Patients were followed up for 1081 ± 365 days to determine lead performance. A brief description of the clinical characteristics of the patients enrolled in the study is presented as Table 1. In terms of the tricuspid valve disease involved, most of the patients had moderate to severe organic TR (21) with 4 cases of 20 prosthetic valves involved (3 metallic valves and 1 bioprosthetic valve).

**Table 1:** Clinical characteristics of enrolled patients in the LV lead series

Characteristics		Implant data	
Gender		Status at 2 Years	
Male	10	Alive	20
Female	16	Deceased	6
Age (years) at Implantation	71.7	Duration of Follow‐Up	1081 ± 365 days
Type of Tricuspid Disease		Type of Implant	
Moderate‐Severe Organic TR	21	Single Chamber	12
Prosthetic TVR	1	Dual Chamber	14
Repaired TV	4		
Indication for Implant		Location of Implant	
Sinus Dysfunction	11	Posterolateral cardiac vein	6
Tachy‐Brady Syndrome	4	Posterior cardiac vein	0
Slow AF with High Grade AVB	3	Middle cardiac vein	4
Complete Heart Block	6	Anterior interventricular vein	6
		Lateral cardiac vein	10

In all 26 cases, implantation was initially successful and the majority of the LV leads were implanted in the lateral cardiac vein. Two patients required lead adjustments on day 1 of implantation due to noted lead dislodgement. In the first case, the LV lead was originally implanted into the middle cardiac vein but dislodged. On reassessment, there were high thresholds in the anterior cardiac vein while in the more septal branches lead stability was questionable as the guidewire would freely enter the right ventricle. Finally, the LV lead was re‐implanted into the middle cardiac vein successfully. In the second case, the LV lead was initially implanted into the posterolateral vein. On reassessment, the middle cardiac vein and great cardiac vein branches had poor thresholds and the anterolateral vein could not be cannulated successfully, after much attempts, decision was made to convert to a conventional RV lead due to the lack of a suitable landing site within the coronary veins.

Over the course of clinical follow‐up spanning 1081 ± 365 days (2.96 ± 1 year), one patient developed *methicillin sensitive staphylococcus aureus (MSSA)* bacteraemia due to thrombophlebitis related to an intravenous cannula and required lead extraction. For this patient, a conventional RV lead was implanted after the original infected LV lead was extracted. None of the patients otherwise developed further dislodgement or malfunction of the LV leads during the course of follow‐up. Of the 26 patients followed up in the study, 6 died within the duration of the follow‐up. Of them, 2 died of pneumonia, while the other 4 died in the community and the causes of death were unable to be captured in the study. In the patients with the conventional RV pacemakers, there were a total of 3 mortalities during the course of the follow‐up. Of them, 1 died of congestive cardiac failure while 2 died in the community and causes of death were also unable to be captured in the study. There was no significant difference in mortality or lead complications between the two groups.

Table 2 shows the baseline and procedural characteristics of the case and controls. The controls were matched to the cases for age, gender, LVEF (within 10%) as well as the type of device implanted (single or dual chamber). The primary indications for pacemaker were sinus node dysfunction, complete heart block, slow AF with high grade AV block, and tachy‐brady syndrome in both populations.

**Table 2:** Clinical characteristics of the matched patients from both LV and RV series

	LV pacemaker (n = 26)	RV pacemaker (n = 26)
General characteristics		
Age at implantation	71.7 ± 11.3	71.0 ± 11.5
Gender (male)	10	10
Type of Implant (VVI/ DDD)	12/ 14	12/ 14
LVEF	53.6 ± 9.8	57.6 ± 10.0
Indications for implant		
Sinus node dysfunction	11	8
Tachy‐Brady syndrome	4	2
Complete heart block	6	12
AF with low ventricular rate	3	4

Table 3 shows the lead interrogation data from the time of implant and at subsequent follow‐ups. The pacing capture threshold amplitude and the pacing impedance were higher for LV leads (1.46 ± 0.83 mV and 822 ± 152 ohms) compared to RV leads (0.73 ± 728 ± 215 ohms). Repeated measures ANOVA testing was then conducted for the impedance values measured across time. It was shown that over time the lead impedance remained stable throughout duration of follow‐up in both RV and LV leads (*P* = .196). Over the long‐term follow‐up, the impedance in the LV leads remained higher than that in the RV leads (*P* < .001).

**Table 3:** Electrophysiological parameters after pacemaker implantation

	LV PPM (n = 26)	RV PPM (n = 26)	*P* value
At implant			
Fluoroscopy time	30 min 19 s	7 min 49 s	<.001
Pulse output (V@0.4ms)	1.46 ± 0.83	0.73 ± 0.30	<.001
Pulse width (ms)	0.54 ± 0.30	0.49 ± 0.03	.423
Pacing impedance (Ω)	822.15 ± 151.93	728.08 ± 215.54	.077
R wave amplitude (mV)	9.29 ± 5.16	12.10 ± 6.15	.084
6 months follow‐up			
Pulse output (V)	1.23 ± 0.43	0.70 ± 0.17	<.001
Pulse width (ms)	0.64 ± 0.37	0.40 ± 0.02	.003
Pacing impedance (Ω)	784.79 ± 212.72	554.69 ± 103.40	<.001
R wave amplitude (mV)	12.57 ± 7.73	14.22 ± 8.30	.471
Percentage ventricular paced (%)	31.4% ± 36.7%	47.9% ± 41.7%	.135
Last follow‐up			
Time to follow‐up	1081 ± 365	1529 ± 443	<.001
Pulse output (V)	1.21 ± 0.47	0.74 ± 0.19	<.001
Pulse width (ms)	0.71 ± 0.37	0.42 ± 0.12	<.001
Pacing impedance (Ω)	718.42 ± 260.62	539.69 ± 135.87	.003
R wave amplitude (mV)	12.10 ± 6.64	11.28 ± 7.25	.672
Percentage ventricular paced (%)	40.9% ± 42.1%	52.9% ± 42.8%	.309
Final outcomes			
Lead dysfunction/ revision	3	0	.235
Mortality	6	3	.465

One significant observation was that the fluoroscopy times for the LV pacemakers were significantly longer than that for the RV pacemakers. However, it should be noted that excluding the first 3 cases which took a longer time perhaps due to the learning curve for the LV pacemaker implantation, the average fluoroscopy time for an LV pacemaker was 23min 32s.

Follow‐up transthoracic echocardiography was performed routinely pre‐device implantation and at around 2 years post implantation and findings are shown in Table 4. The LVEF in general improved after the device implantation for LV lead patients. During the duration of the follow‐up, two patients underwent further interventions to the tricuspid valve; one had a bioprosthetic tricuspid valve replacement and one had a tricuspid valve annuloplasty. For the rest of the patients, the severity of TR improved in 5 patients, was unchanged in 12 patients and worsened in 2 patients. Among the patients with RV leads implanted, there was an overall worsening of TR severity. TR severity improved in 2 patients, was unchanged in 13 patients, but worsened in 11 patients.

**Table 4:** Transthoracic echocardiography before and after pacemaker implantation

	LV PPM (n = 26)			RV PPM (n = 26)		
	Pre‐implant	2‐year post	*P* value	Pre‐implant	2‐year post	*P* value
Left ventricle						
LVEF	54.2 ± 9.8	58.2 ± 6.9	.118	57.5 ± 10.2	55.5 ± 9.7	.444
LVID(d)	47.4 ± 7.6	45.4 ± 7.6	.404	48.4 ± 7.5	46.3 ± 7.0	.307
LVID(s)	31.8 ± 7.1	30.6 ± 7.0	.672	32.6 ± 6.4	30.9 ± 7.2	.386
Median MR	Mild‐moderate	Mild‐moderate	.613	Mild	Mild	.124
LA size	46.0 ± 10.7	48.0 ± 11.3	.539	45.4 ± 9.7	46.7 ± 9.8	.632
Right ventricle						
RVD(d)	22.6 ± 7.8	27.0 ± 10.2	.217	14.5 ± 11.3	19.9 ± 8.0	.090
Median TR	Moderate	Moderate	.205	Mild	Moderate	.048
Cases in AF	13	13	.658	12	12	.783

## 4 DISCUSSION

This report shows the overall safety and feasibility of the LV pacing approach in patients with tricuspid valve disease in whom a right ventricular pacemaker lead was avoided. Our findings demonstrate acceptable long‐term reliability with no late lead dislodgements, malfunctions, and minimal adverse clinical events.

Worsening TR post device implantation may be especially problematic in patients with preexisting tricuspid valve disease or prior tricuspid valve surgery. There have been various case series with patients in whom transvalvular leads were contraindicated, most frequently in patients with a mechanical tricuspid valve and traditionally an epicardial system was implanted. However, epicardial leads typically require sternotomy or thoracotomy for insertion and tend to have lower reliability and higher pacing thresholds than their endocardial counterparts.^9^ With the advent of coronary sinus lead placement, permanent leads can now be delivered transvenously for the purpose of ventricular pacing in these patients without the need to cross the tricuspid valve.^10^ However there remain concerns regarding the feasibility of ventricular pacing via the coronary sinus as complications can include diaphragmatic stimulation, coronary sinus dissection and lead displacement.^13^ Particularly, due to anatomical considerations and a lack of active fixation with all CS leads bar one currently, lead stability had been a concern over the years.

### 4.1 LV lead parameters remain stable

However, in this study we found that over 3 years’ follow‐up, there were no cases of lead malfunction or failure among our LV paced cases and only one case did develop MSSA bacteraemia requiring lead extraction. We also found that while LV pacing thresholds were higher than the conventional pacing thresholds as in RV pacing, in keeping with prior reports.^14^, ^15^ Overall, the LV capture thresholds remained stable and all were below 2.0 V. Furthermore, the relatively higher pacing impedance in LV leads would also mitigate their higher pacing thresholds in relation to battery drain and hence the impact on battery longevity may not be as significant. It is also noteworthy that we did not have any problems with diaphragmatic capture in this case series after the pacing parameters were optimized before discharge after device implantation.

### 4.2 Longer procedural times

In this present series the average fluoroscopy time when implanting an LV lead was significantly longer than the corresponding RV pacemaker procedures. This reflects the additional steps of accessing the coronary sinus as well as the greater technical challenge of the procedure which is contributed by the variability in coronary venous anatomy and the lack of muscular trabeculae that aid in anchoring a lead in the RV.^14^ In our experience, the first three cases had a significantly longer fluoroscopy time of longer than an hour which is expected given the initial learning curve. Excluding these cases, the average fluoroscopy time of implanting an LV lead was around 23 minutes and comparable to that reported by other studies as well.^15^, ^16^ However, the overall success rate in our experience does show that in trained hands, despite the relative difficulty of the procedure, the procedure remains one that is safe and feasible.

In terms of selecting the site for lead implantation, the mid‐lateral wall has traditionally been the preferred site to maximize cardiac resynchronization therapy. On the other hand, placement of a ventricular lead in the coronary venous system as the sole ventricular pacing electrode affords more flexibility in choice of target veins, for example, in the middle cardiac vein or anterior interventricular vein, and with more apical positioning to ensure reliable pacing support during long‐term follow‐up. It is also important to ensure that that the heel of the lead along the right atrial course does not prolapse into the TV apparatus potentially impairing valve function. Furthermore, positioning the left ventricular pacing lead in the distal anterior cardiac vein is often technically easier than in a lateral or postero‐lateral branch, with less risk of phrenic nerve capture.

### 4.3 No change in LVEF

Over the course of the follow‐up, there was no significant deterioration in the LVEF in patients with either an RV or LV lead. However, there was a non‐significant numerical improvement in patients with an LV lead while those with an RV lead had a slight decrease. This is in keeping with previous studies that long‐term right ventricular pacing may be deleterious to long‐term cardiac function.^8^ Studies have also shown that LV pacing may have a similar beneficial impact as biventricular pacing on LV reverse remodelling, 6 min walk distance, aerobic exercise performance, and prevention of adverse clinical outcomes like heart failure hospitalization or death.^17^ However, the sample size of our study is too small and whether single site LV pacing via the coronary sinus offers a superior single‐site ventricular pacing with respect to long‐term clinical outcomes remain uncertain.

Though not a primary endpoint of the study, while monitoring the echocardiographic parameters of the followed cases, we noted that there was a general worsening of tricuspid valve function in 11 out of 26 patients with the RV lead. Notably, there was a concomitant increase in the RV diastolic diameter in these same patients with a significant increase in the RV size from 14.5 to 19.5 mm. This reinforces the point that an RV pacing lead is not always benign and can lead to long‐term detrimental consequences. On the other hand, TR was improved or unchanged in 20 out of 26 patients who had LV pacing, while it deteriorated in 2 patients and 2 more underwent tricuspid valve surgery and 2 were deceased before further follow‐up echocardiography could be done. In the two patients who had worsening of their TR, in one case there was noted prolapse of the LV lead across the tricuspid valve while in the other, the LV lead had already been replaced by the time the repeat echocardiogram was done. This is pertinent as these patients already have preexisting tricuspid valve disease and this suggests that LV pacing may reduce the likelihood of worsening TR. At the same time, the fact that there were preexisting tricuspid valve abnormalities in these patients makes it uncertain whether the tricuspid valve was more affected in the RV pacing group compared to the LV pacing group as these groups are not directly comparable. Nonetheless, it suggests that patients who are at risk of worsening TR, especially those with preexisting tricuspid valve disease can be considered for LV pacing in order to reduce the likelihood of exacerbating it.

### 4.4 Limitations

A small retrospective analysis like ours has inherent limitations in ascertaining cause and effect and this does apply to our study. A further limitation is that we were unable to determine the causes of death for the patients who passed on in the community and whose causes of death were not captured in the national electronic health records. For this study, we were unable to access the national death registry due to patient privacy laws. Moving forward, a prospective study evaluating LV versus RV pacing for routine pacemaker implants may be useful in confirming and further elaborating on the findings of this study.

With the availability of leadless pacemakers recently, it could be argued that LV pacing to prevent tricuspid valve issues is now less important. However, current leadless systems can only pace the ventricle and their whole life performance is still unknown. In younger patients who are likely to live longer than the expected battery longevity, the ability to and safety of implanting multiple leadless pacemakers is still not clear. Hence, there is still a role for LV pacing systems in such patients with tricuspid valve disease who require ventricular pacing, especially in the form of dual chamber pacemakers.

## 5 CONCLUSION

LV pacing with a lead through the coronary venous system is a safe alternative with good long‐term reliability in patients who require pacing but who have contraindications to placement of a lead across the tricuspid valve.

## CONFLICT OF INTERESTS

The authors declare no conflict of interests for this article**.**
